# Trigeminal Cardiac Reflux During Le Fort I Osteotomy: A Case Report

**DOI:** 10.7759/cureus.61665

**Published:** 2024-06-04

**Authors:** Hissah Alshalawi, Bader Fatani, Mohammed Alotaibi, Zayed A Assiri

**Affiliations:** 1 Dentistry, College of Dentistry, King Saud University, Riyadh, SAU; 2 Maxillofacial Surgery, Prince Sultan Military Medical City, Riyadh, SAU; 3 Oral and Maxillofacial Surgery, Ministry of Health, Aseer Central Hospital, Abha, SAU

**Keywords:** orthognathic surgery, oculo-cardiac reflex, trigeminocardiac reflex, maxillo-facial, lefort 1 osteotomy, trigeminocardiac reflex trigeminal nerve

## Abstract

The trigeminocardiac reflex (TCR) is marked by significant cardiovascular reactions, such as bradycardia and asystole, triggered by trigeminal nerve stimulation. It is described as a brief episode of bradycardia, hypotension, or even cardiac arrest resulting from trigeminal nerve stimulation. The exact cause of TCR is not yet fully understood, but it is believed to involve the release of neurotransmitters, including acetylcholine, and the involvement of central neuronal integration. In this case report, we present an occurrence of trigeminal cardiac reflux during a Le Fort I osteotomy procedure in a patient with no medical issues.

## Introduction

The interaction between the trigeminal nerve, a major cranial nerve responsible for facial sensation, and the cardiac reflex, a vital autonomic response regulating heart rate and rhythm, has been a focus of medical research [1]. As Tarabanis et al. [2] reveal, the activation of the trigeminal nerve and cardiac reflex during specific medical operations causes the trigeminocardiac reflex (TCR), a phenomenon in which trigeminal nerve stimulation causes significant cardiovascular reactions such as bradycardia and asystole. As such, Arora and Lee [3] posit that the dynamic relationship between these neurological pathways emphasizes the need to know the triggers and processes of TCR, especially in craniofacial surgery, where precise manipulations near the trigeminal nerve are prevalent. 

The trigeminocardiac reflex, described as a brief, self-limiting episode of bradycardia, hypotension, or even cardiac arrest caused by trigeminal nerve stimulation, occurs as a result of complicated pathophysiological pathways [4]. While the precise etiology is unknown, the release of neurotransmitters such as acetylcholine, as well as the participation of central neuronal integration, have been hypothesized as critical components in the reflex's beginning [5,6]. Bohluli et al. [1,7] found that TCR is triggered by different things, such as manipulating cranial nerves, especially the trigeminal nerve, and different types of maxillofacial surgeries. Moreover, Maharaj et al. [8] state that surgical procedures like Le Fort osteotomies, craniofacial reconstructions, and manipulation of ocular muscles also cause TCR triggers. The occurrence of TCR, as found in Guedes et al.’s and Chowdhury et al.’s studies [5,6], is affected by the complexity of the surgery, individual patient factors, and the attentiveness of the surgical team. 

While the incidence and importance of the TCR in other branches of surgery have only recently been recognized, craniofacial surgery involving osteotomies and soft-tissue manipulation in the region innervated by the mandibular, maxillary, and ophthalmic divisions of the trigeminal nerve has been found to induce the reflex. Additionally, advances in skull base surgery have allowed access to areas such as the cerebellopontine angle, cavernous sinus, and pituitary fossa, where the TCR can occur during procedures such as microvascular decompression surgery of the trigeminal nerve and balloon-compression rhizotomy of the trigeminal ganglion. Although transient cardiovascular changes, including decreases in heart rate and blood pressure, have been observed in these cases, they are usually reversible and do not result in significant postoperative complications [9].

Furthermore, procedures such as Le Fort 1 osteotomy, which involves manipulations along the trigeminal nerve, provide a considerable risk of evoking TCR in the context of craniofacial surgery [8]. TCR has significant consequences in such procedures, stressing the need for close monitoring and precise approaches to avoid reflex initiation. Hence, Bohluli et al. [7] posit that preventive strategies such as controlled anesthesia and precise surgical procedures are critical in avoiding TCR-related problems. besides,  Mhamunkar et al. [10] claim that these preventative measures are critical protections during maxillofacial procedures, ensuring patient safety and a quick recovery

## Case presentation

**Figure 1:**
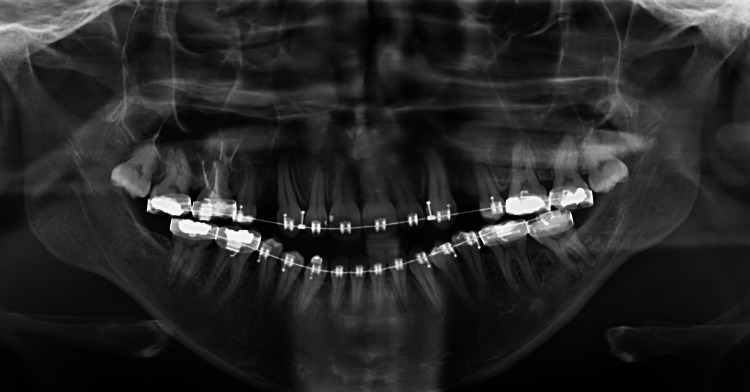
Pre-operative panoramic radiograph. This is a panoramic radiograph of the patient before undergoing the orthognathic surgery which included Le Fort 1 osteotomy, bilateral sagittal split osteotomy, and advancement genioplasty to correct his skeletal and dental disharmony.

A 32-year-old Saudi male patient, non-smoking and unaware of any medical history. The patient is not currently treated with any kind of medication and has no reported allergies. The patient was seen in a combined orthodontic-maxillofacial surgery clinic for dentofacial deformities after being referred by an orthodontist as a case of hypoplastic maxilla that cannot corrected orthodontically alone. The case was prepared orthodontically followed by routine orthognathic preparation including clinical examination, photos, lateral and posterior-anterior cephalometric, orthopantogram then computed tomography. The pre-operative panoramic radiograph is demonstrated in Figure 1.

The decision was made in a combined clinic as a case of a hypoplastic maxilla that was ready for surgery. The patient was planned for Le Fort I osteotomy and bilateral sagittal split osteotomy with advancement genioplasty. After shifting the patient to the operating room, general anesthesia was performed by the anesthesia uneventfully. Regular preparation and steps are made smoothly. The Le Fort I osteotomy was completed, and once downward fracture movement of the maxilla was initiated by applying finger pressure on the maxilla to the proposed direction, the anesthesiologist noted a sudden onset of reduction in heart rate starting from 98 beats per minute to 40th reaching 25 beats per minute. The procedure was held for anesthesia evaluation and Atropine was given from the anesthesia team. The heart rate after that was improved to 71 beats per minute. Following additional manipulation of the maxilla downwards, the anesthesia reported a significant reduction in the heart rate reaching 30 beats per minute. Anesthesia noticed that and corrected it using atropine same previous dose. The down fracture of maxilla already went smoothly at bradycardia onset and there were no additional episodes of bradycardia after that. From the surgical site, there was no bleeding or oozing noted at surgical sites and hemostasis was maintained throughout the whole procedure. The episodes were noted mainly related when pressure on the posterior maxilla was applied at down fracture of the maxilla only, otherwise, there were no episodes of bradycardia.

**Figure 2:**
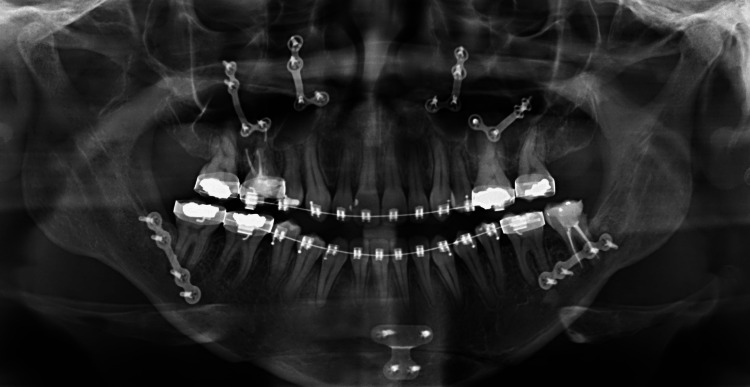
Post-operative panoramic radiograph. This is a panoramic radiograph of the patient after undergoing the orthognathic surgery which included Le Fort 1 osteotomy, bilateral sagittal split osteotomy, and advancement genioplasty.

Two potential differential diagnoses were considered and investigated regarding the sudden onset of the bradycardia. First, anesthetic medication-related bradycardia was ruled out after assessing the administered medications, doses, and frequency as well. Second is the trigeminal nerve-related bradycardia which is caused by trigeminal-cardiac reflux. The procedure was then carried out with careful manipulation of the fractured maxilla. No further episodes were noted in the procedure afterward. The patient was then extubated and shifted to the recovery room and was assessed by a cardiologist through an electrocardiogram and reported no appointed abnormalities with normal sinus rhythm. The patient was then admitted for two days for further assessment with no post-operative bradycardia noticed while admitted to the hospital. The patient denied any previous episodes of bradycardia or even cardiac compliant. The postoperative panoramic radiograph is illustrated in Figure 2.

At the first follow-up appointment one week after discharge, the patient reported that in the postoperative period when using water floss with high pressure on the posterior area of the maxilla, he felt slight dizziness which was improved by stopping the brushing. Also, at first visit follow-up after removing the intermaxillary fixation in the right posterior area, a slight force was directed to remove one of the elastics, the patient had a sudden loss of consciousness with the vitals showing a significant decrease in the heart rate. After careful adjusting of the force of the elastic a sudden recovery of the patient's consciousness was observed without any further episodes. The patient is currently in a follow-up period for assessment and monitoring.

## Discussion

The trigeminal nerve, one of the major cranial nerves responsible for facial sensation, and the cardiac reflex, a crucial autonomic response regulating heart rate and rhythm, have been the focus of extensive medical research in recent years [1]. The interaction between these two systems gives rise to a fascinating phenomenon known as the TCR. The TCR is characterized by significant cardiovascular reactions, such as bradycardia and asystole, triggered by trigeminal nerve stimulation [9]. Understanding the intricacies of this dynamic relationship and unraveling the triggers and mechanisms of TCR is of utmost importance, particularly in the context of craniofacial surgery where precise manipulations near the trigeminal nerve are prevalent [3].

The TCR is described as a transient episode of bradycardia, hypotension, or even cardiac arrest resulting from trigeminal nerve stimulation [4]. The exact etiology of TCR is not yet fully understood, but it is believed to involve the release of neurotransmitters, including acetylcholine, and the involvement of central neuronal integration [5,6]. Several triggers have been identified for TCR, including manipulations of cranial nerves, particularly the trigeminal nerve, and various types of maxillofacial surgeries [1,7]. For example, procedures such as Le Fort osteotomies, craniofacial reconstructions, and the manipulation of ocular muscles have been associated with the occurrence of TCR [8]. 

While the incidence and significance of TCR in other surgical branches have only recently been recognized, craniofacial surgery involving the mandibular, maxillary, and ophthalmic divisions of the trigeminal nerve has been found to induce the reflex [9]. Furthermore, advances in skull base surgery have expanded the possibilities of accessing areas such as the cerebellopontine angle, cavernous sinus, and pituitary fossa, where TCR can occur during procedures like microvascular decompression of the trigeminal nerve and balloon-compression rhizotomy of the trigeminal ganglion [9]. Although transient cardiovascular changes, including decreases in heart rate and blood pressure, have been observed during these cases, they are typically reversible and do not result in significant postoperative complications. 

Given the considerable risk of TCR induction in craniofacial surgery, preventive strategies have become paramount in avoiding TCR-related issues [7,10]. Controlled anesthesia and precise surgical techniques are critical measures employed to mitigate the occurrence of TCR and ensure patient safety. These strategies involve careful monitoring and management of anesthesia depth, maintaining adequate blood pressure and heart rate, and minimizing trigeminal nerve stimulation during surgical procedures [10]. Additionally, the administration of anticholinergic medications, such as atropine, may be considered to counteract the parasympathetic response associated with TCR [7].

The prevention and management of TCR during craniofacial surgery require a comprehensive understanding of the reflex and its implications. Surgeons must be vigilant in recognizing potential triggers and taking appropriate measures to minimize their impact. Moreover, an interdisciplinary approach involving anesthesiologists, neurosurgeons, and craniofacial surgeons is essential to ensure coordinated efforts in preventing and managing TCR [10].

In relation to the current literature, our case report demonstrated unusual postoperative symptoms. During the first follow-up visit after removing the intermaxillary fixation in the right back area, when a gentle force was applied to remove one of the elastics, the patient experienced a sudden loss of consciousness with a notable decrease in heart rate. By carefully adjusting the pressure of the elastic, the patient quickly regained consciousness without recurring episodes. The patient is currently under observation during a follow-up period for assessment and monitoring.

## Conclusions

In this report, we illustrate a case of trigeminal cardiac reflux that occurred while performing Le Fort I osteotomy in a medically free patient. A careful manipulation of the maxilla throughout the Le Fort I osteotomy procedure is essential to avoid bradycardia caused by trigeminal nerve reflux. Moreover, assessing the patient in the postoperative period is critical especially while adjusting the elastics to prevent any further incident of trigeminal cardiac reflux. While the incidents of such cases are not significantly reported in the literature. However, the surgeon should carefully assess each case independently to prevent any further complications.
